# Relationships within *Mcneillia* Indicate a Complex Evolutionary History and Reveal a New Species of *Minuartiella* (Caryophyllaceae, Alsinoideae)

**DOI:** 10.3390/plants11162118

**Published:** 2022-08-15

**Authors:** Daniele De Luca, Emanuele Del Guacchio, Fabio Conti, Duilio Iamonico, Paolo Caputo

**Affiliations:** 1 Department of Biology, University of Naples Federico II, Via Foria 223, 80139 Naples, Italy; 2 School of Biosciences and Veterinary Medicine, University of Camerino, San Colombo-Via Provinciale, 67021 Barisciano, Italy; 3 Department PDTA, University of Rome Sapienza, Via Flaminia 72, 00196 Rome, Italy; 4 Botanical Garden of Naples, University of Naples Federico II, Via Foria 223, 80139 Naples, Italy

**Keywords:** amphiadriatic taxa, Caryophyllales, Mediterranean mountain flora, *Minuartia*, reticulate evolution, taxonomy

## Abstract

The genus *Mcneillia* has been recently segregated from *Minuartia* L. based on molecular results, also supported by morphology. However, to date, a comprehensive study on the phylogenetic relationships within this genus is lacking. In this paper, we provide a multigene phylogeny of all the species and subspecies of *Mcneillia* employing two nuclear and six chloroplast markers. We documented extensive gene flow between taxa, sometimes separated at specific rank. In addition, *Mcneillia* as currently circumscribed, is not monophyletic. In fact, *Mcneillia graminifolia* subsp. *brachypetala*, strictly endemic to Greece, truly belongs to *Minuartiella*, a genus otherwise limited to South-West Asia. Moreover, even after removal of this taxon, our results do not support the monophyly of the taxa included in *M. graminifolia* s.l., the most variable and widespread species of the genus. Further controversial subspecies of *Mcneillia graminifolia*, i.e., subsp. *hungarica* and subsp. *rosanoi*, are shown to deserve taxonomic recognition as separate species, whereas *Mc. moraldoi* is not distinct at specific rank. In addition, *Mc. saxifraga* subsp. *tmolea* is here regarded as a further distinct species. A consistent taxonomic treatment is therefore proposed with six new combinations and nomenclatural notes, providing the necessary typifications.

## 1. Introduction

In the last decades, the application of DNA data to plant taxonomy has allowed an accurate understanding of the patterns of descent and, therefore, has fostered taxonomic revision in virtually all groups of plants. Molecular techniques, which are increasingly easily and generally available, have allowed phylogenetic investigation also in speciose genera (e.g., [1,2,3,4,5]) with consequent taxonomic revision as well (e.g., [6,7,8]). Within Caryophyllaceae, phylogeny has been investigated, for example, in *Acantophyllum* C.A.Mey. [9], *Arenaria* L. [10], *Atocion* Adans. [11], *Cherleria* L. [12], *Facchinia* Rchb. [13], *Habrosia* Fenzl [14], *Heliosperma* [15], *Petrocoptis* A.Braun ex Endl. [16], *Polycarpon* Loefl. [17,18], *Pseudocerastium* C.Y.Wu, X.H.Guo & X.P.Zhang [19], *Pseudostellaria* Pax [20], *Silene* L. [21,22,23], *Stellaria* L. [24,25], *Viscaria* Bernh. [11], and *Minuartia* Loefl. s.l. [26].

**Figure 1:**
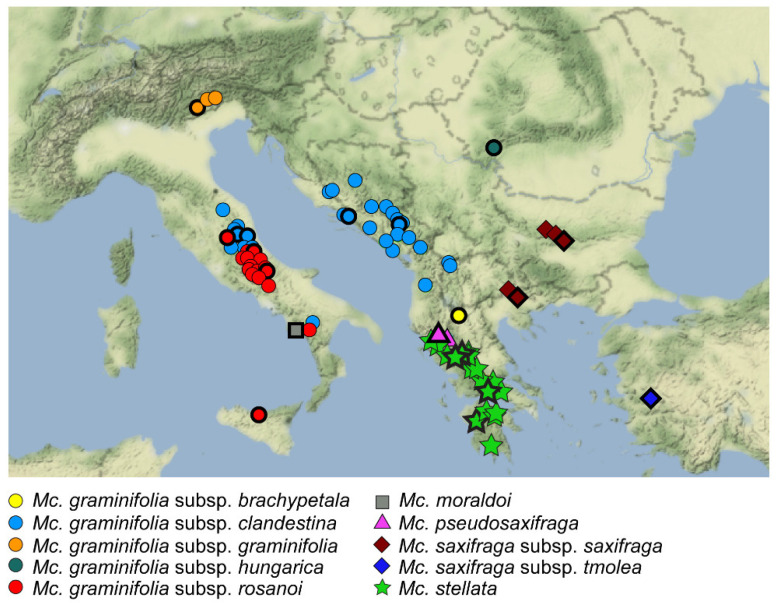
Distribution of the species and subspecies of *Mcneillia*. Symbols marked with a thick black line indicate the localities sampled in the present study.

Dillenberger and Kadereit [26] documented rampant polyphyly in *Minuartia* as traditionally conceived, revealing that the main diagnostic characters for the genus (i.e., presence of three styles and three fruit valves) are indeed plesiomorphic. Even subgeneric ranks (e.g., *Minuartia* subg. *Minuartia*) were often non-monophyletic and included clades related to other genera. As a consequence, the said authors limited *Minuartia* to sects. *Plurinerviae* McNeill and *Minuartia*, transferring the other species to different genera, monophyletic as far as possible. In particular, *Minuartia* sect. *Lanceolatae* (Fenzl) Graebn. Ser. *Graminifoliae* Mattf. was segregated into *Mcneillia* Dillenb. & Kadereit, distributed from Italy to Anatolia [26]. This small genus includes evergreen perennial herbs (sometimes suffruticose) up to 20(−40) cm tall, which are mainly densely caespitose to pulvinate, are often with glandular hairs, especially on the shortened peduncles, with terete stems, have lanceolate to linear (rarely oblanceolate) leaves (the cauline ones similar to the bracts), have flowers with petals white, which are erect or suberect at anthesis, obovate and cuneate to the base, and are usually longer than sepals, which are 5-7(-9)-veined; they generally occur in rocky places especially on limestone (less frequently on granites, gneiss or conglomerates), at 600–2400 m.a.s.l. ([26,27,28,29,30], pers. obs.). At present, the genus (in its most diversified and complete treatment, see [31]), includes five species [26,32] (for convenience’s sake, hereafter the generic names *Mcneillia*, *Minuartia*, and *Minuartiella* will be abbreviated respectively as “*Mc*.”, “*Mn*.”, and “*Ml.*”): *Mc. graminifolia* (Ard.) Dillenb. & Kadereit [≡ *Mn. graminifolia* (Ard.) Jáv], distributed from Italy (including Sicily), through the Balkan peninsula to South-Eastern Romania; *Mc. moraldoi* (F.Conti) Dillenb. & Kadereit (≡ *Mn. moraldoi* F.Conti), narrowly endemic to South-Western Italy on Mount Gelbison (Campania region); *Mc. pseudosaxifraga* (Mattf.) Dillenb. & Kadereit [≡ *Mn. pseudosaxifraga* (Mattf.) Greuter & Burdet], endemic to Northern Greece (Mt. Pindhos) and Albania (Mt. Nemercka); *Mc. saxifraga* (Friv.) Dillenb. & Kadereit [≡ *Mn. saxifraga* (Friv.) Graebn.], growing from Bulgaria and Northern Greece to Turkey; and *Mc. stellata* (E.D.Clarke) Maire & Petitm. [≡ *Mn. stellata* (E.D.Clarke) Maire & Petitm.], growing on limestone rocks in Greece and Albania [33]. Within the polymorphic *Mc. graminifolia*, the typic subspecies is endemic to the Central-Eastern Italian Alps; subsp. *brachypetala* (Kamari) Dillenb. & Kadereit [≡ *Mn. graminifolia* subsp. *brachypetala* Kamari] is restricted to Mt. Boutsi in Northern Greece near the border with Albania; subsp. *clandestina* (Port.) Dillenb. & Kadereit is amphi-Adriatic (Italian peninsula, Croatia, Albania, North Macedonia), subsp. *hungarica* (Jáv.) F.Conti & Bartolucci [≡ *Mn. graminifolia* subsp. *hungarica* Jáv] is endemic to Mt. Arjana (South-Eastern Romania); and subsp. *rosanoi* (Ten.) F.Conti, Bartolucci, Iamonico & Del Guacchio occurs in the Apennines and in Sicily [32,33,34]. Infraspecific taxa have also been recognized in *Mc. saxifraga*, with subsp. *saxifraga* occurring in Bulgaria and Northern Greece and subsp. *tmolea* (Mattf.) Dillenb. & Kadereit endemic to Mt. Tmolus (Western Anatolia, Turkey) (Figure 1).

The systematics of the group, however, are far from being entirely accepted. A synopsis of the most relevant taxonomic treatments is provided in Table 1. In addition, the relationships between the taxa are still largely speculative (e.g., [27]), and the phylogeny almost unknown; as at present only four species (mostly with single specimens) have been investigated by molecular methods [26]. In this study, we infer nuclear and plastid phylogenies of genus *Mcneillia* involving all its species and subspecies across their geographic distributions. This paper aims at verifying whether the genus is monophyletic, and whether the current taxonomic treatment correctly depicts phylogeny. As a contribution for the systematics of Alsinoideae, we also propose a consistent taxonomic treatment with typification of the taxa under study.

## 2. Results

### 2.1. ITS Phylogeny

**Figure 2:**
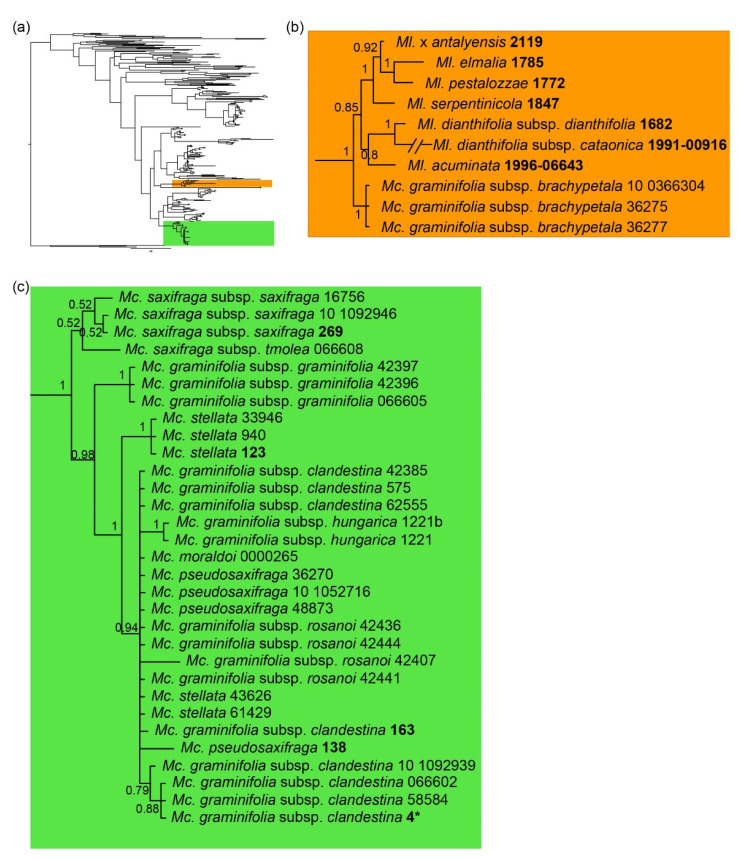
Consensus tree from a Bayesian analysis employing ITS DNA data. Sequences are from the specimens employed by Dillenberger and Kadereit [26], Koç et al. [35], and the present study. (**a**) Outline of the phylogeny of *Minuartia* s.l. (290 terminals); (**b**) *Minuartiella* clade; (**c**) *Mcneillia* clade. In bold: voucher codes of sequences from the literature (see text for references); the sequence labelled as *Mc. graminifolia* subsp. *clandestina* 4 (here marked with an asterisk) was reported by Dillenberger & Kadereit [26] as “*Minuartia graminifolia* 4”, but reidentified since.

The consensus tree from the Bayesian analysis conducted using the 290-taxa alignment of our ITS sequences, plus the ones by Dillenberger and Kadereit [26] and those by Koç et al. [35], confirmed that genus *Mcneillia* is not monophyletic as presently circumscribed (Figure 2a) and indicated that the clade including the three specimens of *Mc. graminifolia* subsp. *brachypetala* (*p.p.* = 1) is in the *Minuartiella* clade (*p.p.* = 1), sister to all the other taxa of this genus. The other taxa of *Minuartiella* formed a monophyletic group (*p.p.* = 0.85), further divided into two clades (Figure 2b), one including *Ml. acuminata* and two subspecies of *Ml. dianthifolia* (*p.p.* = 0.80) and the other including *Ml. elmalia* (Aytaç) Dillenb. & Kadereit, *Ml. pestalozzae* (Boiss.) Dillenb. & Kadereit and *Ml. serpentinicola* Koç & Hamzaoglu, plus the hybrid *Ml.* × *antaliyensis* (Parolly & Eren) Koç & Hamzaoglu (*p.p.* = 1).

The other 31 ITS sequences belonging to *Mcneillia* (Figure 2c), which encompassed both ours and those by Dillenberger and Kadereit [26], were included in a single clade (*p.p.* = 1). This poorly resolved group was first divided in two clades, one of which included the specimens of both subspecies of *Mc. saxifraga* (*p.p.* = 0.52) and the other the remaining samples (*p.p.* = 0.98). Within this clade, the group of the three specimens of *Mc. graminifolia* subsp. *graminifolia* resulted sister to the clade including the remaining taxa (*p.p.* = 1). The latter clade showed a group including the three specimens of *Mc. stellata* from Southern Greece (KF737423.1 from Dillenberger & Kadereit [26]; 940 and 33946 from the present study) in a sister group relationship (*p.p.* = 1) to a largely unresolved clade (*p.p.* = 0.94) including *Mc. graminifolia* subsp. *clandestina*, subsp. *hungarica*, subsp. *rosanoi*, *Mc. moraldoi*, *Mc. pseudosaxifraga,* and the other specimens of *Mc*. *stellata* from Northern Greece. Within the latter clade, the samples of subsp. *hungarica* grouped together (*p.p.* = 1), and also some specimens of subsp. *clandestina* from the Balkans formed a clade (*p.p.* = 0.79). In this regard, the sequence KF737501 from Dillenberger and Kadereit [26] was originally labeled by them as “*Minuartia graminifolia* 4”; however, after examination of the voucher specimen (authors’ obs.), we found that it was representative of *Mc. graminifolia* subsp. *clandestina* from Bosnia (see Figure 2c).

### 2.2. Characteristics of the Final Dataset

The nuclear alignment (without *Mc. graminifolia* subsp. *brachypetala*) (Data S1) was composed of 27 taxa and 1303 characters (ITS1, 5.8S and ITS2: 852 bp; ETS: 451 bp) while the chloroplast one (Data S2), resulting from the concatenation of six chloroplast regions, was of 3,265 characters (*rpo*C1: 462 bp; *rps*16 intron: 799 bp; *trn*L-*trn*F: 418 bp; *trn*H-*psb*A: 370 bp; *rpl*32-*trn*L: 574 bp; *rps*16-*trn*Q: 642 bp) and the same number of taxa. Amplification was not successful in some individuals for the following markers: ETS (no. 16756 in C and APP no. 63095), *rps*16 intron (FI no. 066605), and *rpl*32-*trn*L (no. 16756 in C and APP no. 42436). We also amplified and sequenced a pseudogene of the ITS, ~300 bp long, in all three samples of *Mc. graminifolia* subsp. *graminifolia* corresponding to 18S (1–32 bp)–26S (33–303) and another, ~190 bp long, corresponding to ITS1, in sample APP no. 42444 of subsp. *rosanoi*. All sequences were deposited to DDBJ (including the ETS and chloroplast markers of *Mc. graminifolia* subsp. *brachypetala* generated in this study but not utilized in the phylogenetic inference). The best-fitting nucleotide evolutionary models computed for each marker were as follows: F81 (*rpo*C1; *rpl*32-*trn*L); F81 + G (*rps*16-*trn*Q; *trn*H-*psb*A); GTR (*rps*16 intron); GTR + I (ITS); HKY + G (ETS); HKY + I (*trn*L-*trn*F). The ILD test revealed that the nuclear and chloroplast matrices were significantly incongruent (*p* = 1), and therefore nuclear and chloroplast datasets were not merged.

### 2.3. Nuclear and Chloroplast Phylogenies

The stepping-stone analysis aimed at testing strict and relaxed clock models for both datasets produced clear evidence in favor of the strict clock: for the nuclear dataset, the mean marginal likelihood for the relaxed clock (ln) was −3018.19, whereas the mean marginal likelihood for the strict clock was −2920.93; and for the chloroplast dataset, −5663.00 and −5504.37, respectively.

**Figure 3:**
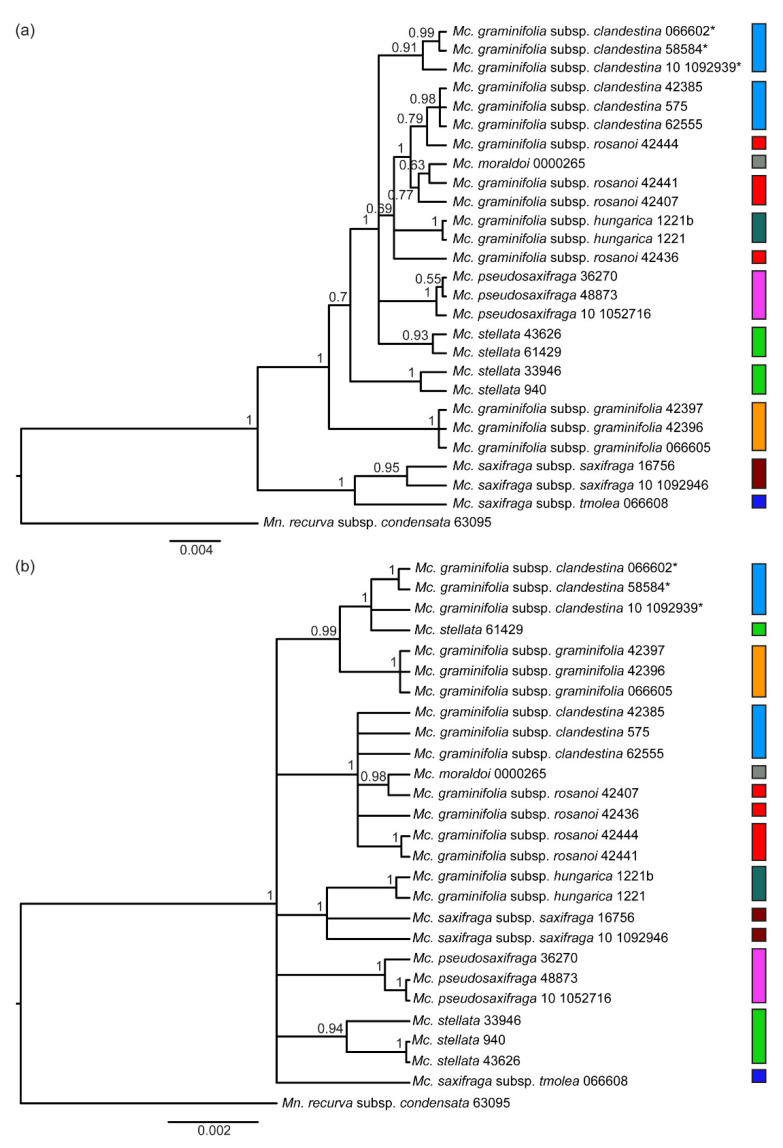
Consensus trees from Bayesian analyses. (**a**): nuclear dataset; (**b**) chloroplast dataset. Color codes are as in Figure 1; the asterisk (*) indicates the Balkan specimens of *Mc. graminifolia* subsp. *clandestina*.

The consensus tree obtained from the Bayesian analysis of the nuclear dataset (Figure 3a), rooted by using *Mn. recurva* subsp. *condensata*, showed two monophyletic groups (each one with *p.p.* = 1). The first was composed by *Mc. saxifraga* subsp. *tmolea* and a branch with the two individuals of subsp. *saxifraga* (*p.p.* = 0.95). The other one included all the other taxa and branched in a first clade with the three samples of *Mc. graminifolia* subsp. *graminifolia* (*p.p.* = 1) and in another one with all the remaining samples (*p.p.* = 0.7). In the latter, a group of the two southern-most specimens of *Mc. stellata* was sister to all the other taxa (each clade with *p.p.* = 1). In this larger, not completely resolved clade, the following groups were recognized: (1) the two northern specimens of *Mc. stellata* (*p.p.* = 0.93); (2) the three samples of *Mc. pseudosaxifraga* (*p.p.* = 1); (3) the Balkan specimens of *Mc. graminifolia* subsp. *clandestina* (*p.p.* = 0.91); and (4) a larger but unsupported group (*p.p.* = 0.69) encompassing all remaining specimens from Italy (i.e., *Mc. graminifolia* subsp. *clandestina* pro parte, subsp. *rosanoi*, and *Mc. moraldoi*) and the two samples of *Mc. graminifolia* subsp. *hungarica*, the latter in a monophyletic unit (*p.p.* = 1). However, all Italian specimens of the fourth group, except a specimen of *Mc. graminifolia* subsp. *rosanoi* from the Gran Sasso massif, formed a well-supported clade (*p.p.* = 1) (Figure 3a).

The consensus tree obtained from the Bayesian analysis of the chloroplast dataset (Figure 3b), rooted as above, presented a less resolved topology and two main instances of incongruence as compared to the nuclear tree: a sister group relationship between *Mc. graminifolia* subsp. *graminifolia* and a clade including the Balkan representatives of *Mc. graminifolia* subsp. *clandestina* plus a specimen of *Mc. stellata* from Northern Greece (*p.p.* = 0.99); and a clade including the specimens of *Mc. saxifraga* subsp. *saxifraga* and those of *Mc. graminifolia* subsp. *hungarica* (*p.p.* = 1), the latter in a monophyletic unit (*p.p.* = 1). Beside these relationships, several other clades occurred and, namely, one including all Italian specimens of *Mc. graminifolia* subspp. *clandestina* and *rosanoi*, as well as *Mc. moraldoi* (*p.p.* = 1); another one composed of the individuals of *Mc. pseudosaxifraga* (*p.p.* = 1); and a third one with three specimens of *Mc. stellata* (*p.p.* = 0.94).

### 2.4. Haplotype Network

**Figure 4:**
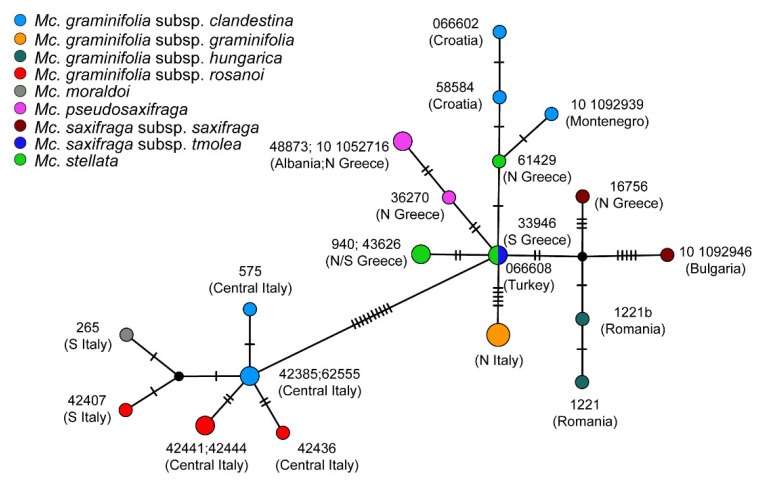
Chloroplast DNA TCS network. Dashes indicate the number of mutations separating haplotypes; the size of nodes is proportional to the number of identical haplotypes.

The TCS network based on chloroplast data showed the occurrence of two main haplogroups separated by 10 mutation steps: one included all the Apennine–Sicilian specimens (i.e., the Italian *Mc. graminifolia* subsp. *clandestina*, *Mc. graminifolia* subsp. *rosanoi*, and *Mc. moraldoi*), and the other all the remaining taxa (Figure 4). The Italian haplotypes resulted as generally separated by one or two mutations, and the haplotypes of the specimen of *Mc. moraldoi* and that of *Mc. graminifolia* subsp. *rosanoi* from Sicily, both from Southern Italy, were more similar to each other than to the other individuals from Central Italy. In the other haplogroup, relationships among haplotypes were more complex: most of them originated from an ancestral haplotype from Turkey and Southern Greece, now found in both *Mc. saxifraga* subsp. *tmolea* and in one specimen of *Mc. stellata*; from this ancestral situation, haplotypes were sorted across (or within) the various taxa in different ways. For instance, the chloroplast haplotypes found in *Mc. graminifolia* subsp. *graminifolia* and *Mc. pseudosaxifraga* originated directly from this ancestral one, and presented five and two to four mutations, respectively. The Balkan specimens of *Mc. graminifolia* subsp. *clandestina* showed a chloroplast haplotype closer to that of a specimen of *Mc. stellata*; this latter species showed a high heterogeneity of chloroplast haplotypes (Figure 4). The haplotypes found in *Mc. graminifolia* subsp. *hungarica* and *Mc. saxifraga* subsp. *saxifraga* originated from an ancestral haplotype, not sampled or possibly extinct; the former taxon resulted separated by the ancestral haplotype by one-two mutations. The two specimens of *Mc*. *saxifraga* subsp. *saxifraga* from two different localities showed very different haplotypes, each separated from the ancestral/unsampled one by three or five mutations (Figure 4).

## 3. Discussion

One of the most remarkable results of the investigations above is that *Mcneillia*, as currently circumscribed, is not monophyletic. In fact, one of the subspecies of *Mc. graminifolia*, i.e., subsp. *brachypetala*, is actually a species of *Minuartiella*.

*Mcneillia graminifolia* subsp. *brachypetala* is a very rare plant, known only for Mt. Boutsi. According to the protologue [36] (p. 190), the most relevant feature of this subspecies is the shortness of the petals (from which the chosen epithet, cf. also [33] (p. 424)). Indeed, Dillenberger and Kadereit [26] indicate that *Mcneillia* differs from *Minuartiella* chiefly on account of its petals longer than sepals and obovate. In addition, *Mc. graminifolia* subsp. *brachypetala* has bracts with a very broad membranous margin reaching the apex [36] (p. 190), and a rigid, slender habit with angulate stems (authors’ obs.). These features are absent in *Mcneillia*, whereas they are diagnostic for *Minuartiella* (cf. *Minuartia* ser. *Dianthifoliae* in [27,28,37]). Finally, *Mc. graminifolia* subsp. *brachypetala* grows in rocky pastures, similarly to various species of *Minuartiella* (see e.g., [35]), and it is not a true chasmophyte as other *Mcneillia* taxa generally are [33]. On account of its phylogenetic position within *Minuartiella* (Figure 2) and geographical isolation, *Mc. graminifolia* subsp. *brachypetala* deserves specific rank. Among *Minuartiella* taxa, it resembles *Ml. dianthifolia* (Boiss.) Dillenb. & Kadereit subsp. *kurdica* (McNeill) Dillenb. & Kadereit (not investigated yet by molecular techniques), on account of its glandular pubescence and densely caespitose habit; but it differs by petal shape (ovate–lanceolate vs. ovate) and length (more than 5 mm vs. less than 5 mm), leaf shape (linear–lanceolate vs. triangular) and number of flowers per inflorescence (3–6 vs. 2–3(4)). It is also similar to *Ml. dianthifolia* subsp. *dianthifolia* (which sometimes shows petals even longer than sepals), but chiefly differs by habit (dense vs. lax) and indumentum (glandular pubescent vs. glabrescent). For these reasons, we propose to transfer *Mc. graminifolia* subsp. *brachypetala* to *Minuartiella*, raising it to species level. In this way, *Mcneillia* becomes a monophyletic unit.

Concerning *Mcneillia* s.s. (as circumscribed just above), we observe that the chloroplast phylogeny is less resolved than the nuclear one and it exhibits two main areas of incongruence, consistent with the geographic distribution of the taxa in study. The first incongruence is the sister relationships between *Mc. graminifolia* subsp. *graminifolia* from the Italian Dolomites (not far from the borders with Slovenia and Croatia) and the Balkan representatives of the subsp. *clandestina* in the chloroplast tree, against the occurrence of *Mc. graminifolia* subsp. *graminifolia* in a sister relationship to all the remaining taxa (barring *Mc. saxifraga* s.l.) in the nuclear tree. In second place, the positions in the two trees of *Mc. graminifolia* subsp. *hungarica* (Romania) and *Mc. saxifraga* subsp. *saxifraga* (Bulgaria and Northern Greece) are notably different. A strong influence of geography is also evident from the distribution of chloroplast haplotypes in the TCS network: not only a sharp separation occurs between a western (Apennine–Sicilian) haplogroup and an eastern one, but the eastern haplogroup includes different haplotypes sorted more coherently with geography than with taxonomy. *Mcneillia stellata*, probably because of its fragmented distribution, has retained high chloroplast variability, partly shared with *Mc. saxifraga* subsp. *tmolea* and not homogeneously distributed within the species. In addition, we can hypothesize that the eastern haplogroup started from a probable Turkish/Southern Greek ancestor, and successively the haplotypes migrated northward, likely in different times; however, a reliable phylogeographic reconstruction of these pathways is not possible. The combination of nuclear/chloroplast incongruence and strong geographical influence on chloroplast DNA topologies is rather common in numerous plant groups (e.g., [38,39]), including several Caryophyllaceae (e.g., [15,40,41]) and has been often interpreted as the consequence of chloroplast capture. This latter event, which may have originated by sympatry in refugia during Pleistocenic glaciations, may be invoked to justify some of the incongruent patterns. Chloroplast capture may have occurred in an Alpine–Dinaric refugium, involving populations of the stem lineages of *Mc. graminifolia* subsp. *graminifolia* and subsp. *clandestina*; and in a southern Carpathian refugium, involving populations of the stem lineages of *M. saxifraga* and *M. graminifolia* subsp. *hungarica*. For these reasons, in the following discussion and the consequent taxonomic treatment, albeit taking into account both trees, we paid attention to the fact that the plastid tree is severely afflicted by geography.

Regarding *Mc. saxifraga* s.l., the nuclear tree fully supports monophyly. Both subspecies included in this taxon share unique features within the genus (e.g., the foliaceous bracts without scarious margin, the direction of outer sepal veins, the number of leaf veins) [28,29] and occupy the eastern-most distribution area of *Mcneillia*. Their ecology has not been thoroughly studied, but, differently from most other *Mcneillia* taxa, they grow on metamorphic rocks ([36]; authors’ obs.). In addition, the nuclear tree confirms the less derived position of *Mc. saxifraga* within the genus, as indicated by Mattfeld [27]. Based on these considerations, *Mc. saxifraga* s.l. may be taxonomically segregated from the remaining taxa. However, we refrain from introducing an infrageneric division within *Mcneillia*, considering the substantial morphological uniformity of this small genus, and also taking into account that the plastid tree shows a strict relationship between *Mc. graminifolia* subsp. *hungarica* and *Mc. saxifraga* subsp. *saxifraga*.

The Turkish taxon *Mc. saxifraga* subsp. *tmolea* has “some claim to recognition at specific rank” [28] and remarkably differs from the European subspecies by several characters such as narrower cauline leaves, up to 2 mm vs. 3–4 mm wide, longer sepals up to 7–8 mm vs. 5–6 mm long. It has not been found elsewhere than in its *locus classicus* (very far from localities of subsp. *saxifraga*). Moreover, the plastid network indicates a different and separate position; and according to the nuclear tree, the branch length separating the two taxa is longer than those separating widely accepted species. Therefore, we propose to raise *Mc. saxifraga* subsp. *tmolea* to specific rank.

Another result of our study is the evident polyphyly of *Mc. graminifolia* (also after excluding subsp. *brachypetala*). In fact, the autonymic subspecies is completely separated, at least in terms of the nuclear DNA signal, from the other infraspecific taxa. Indeed, the phylogram in Figure 3a shows that *Mc. graminifolia* subsp. *graminifolia* is sister to all the other taxa of the genus, except *Mc. saxifraga* subspp. *saxifraga* and *tmolea*. If all subspecies of *Mc. graminifolia* are kept within a single species, this species would necessary include all the other taxa, except *Mc. saxifraga* s.l., i.e., also the morphologically very distinct *Mc. pseudosaxifraga* and *Mc. stellata*. Despite several authors synonymizing subsp. *graminifolia* with subsp. *rosanoi* (Table 1), the chloroplast tree (Figure 3b) would suggest a less remote relationship of the former with subsp. *clandestina*; this is not surprising considering their closer geographical proximity. By a morphological standpoint, *Mc. graminifolia* subsp. *graminifolia*, even if similar to subsp. *rosanoi* (on account of indumentum and petal shapes) and to subsp. *clandestina* (on account of the shape and rigidity of leaves), readily differs by its larger sepals and petals. We therefore believe it is fully justified to regard *Mc. graminifolia* s.s. as a distinct species, a relic after glaciations in a small area of the South-Eastern Alps.

All the Central and Southern Italian specimens of *Mcneillia*, regardless of the taxonomic attribution, form a monophyletic unit in the chloroplast phylogeny. This group broadly appears in the nuclear tree as well, except for one specimen attributed to *Mc. graminifolia* subsp. *rosanoi* (APP no. 42436) from Central Italy (within the main Italian range of subsp. *clandestina*), which falls in a polytomy with this clade.

The status of *Mc. graminifolia* subsp. *clandestina* and subsp. *rosanoi* is difficult to interpret. Molecular data underline a distinctiveness of Italian representatives of subsp. *clandestina* (‘Italian clandestina’, hereafter) from their Balkan counterpart (‘Balkan clandestina’, hereafter), despite no relevant morphological differences having been observed. This discrepancy may be interpreted as a secondary contact between two incompletely differentiated allopatric taxa. In this case, we envisage that the ‘clandestina’ lineage arrived one or more times in Italy and hybridized with the ‘rosanoi’ lineage, with consequent backcrossing toward the latter and isolation from the ’Balkan clandestina’. This hypothesis is compatible with the following observations: (a) the aforementioned morphological similarity between most samples of ‘Italian clandestina’ and ‘Balkan clandestina’, except for the leaves of the flowering stems, which are usually at least ½ as long as internodes in Italy and rarely more than ⅓ as long as internodes elsewhere (cf. [29]); (b) this single character is shared by ‘Italian clandestina’ with the subsp. *rosanoi*; (c) ‘Italian clandestina’is mostly limited to the northern sector of Central Apennines, where it is genetically homogeneous, and likely represents also a migration route for its lineage to reach the Peninsula; and (d) scattered southern localities of subsp. *clandestina* (albeit not recently confirmed and often based on poor material not investigated by molecular means) imply, however, that the ranges of subsp. *clandestina* and subsp. *rosanoi* are largely overlapping and gene flow has occurred extensively. Overall, even if *Mc. graminifolia* subsp. *rosanoi* and subsp. *clandestina*, in their typical forms, are well distinguishable on account of several quantitative characters, and chiefly by the indumentum, the quantitative characters are partly overlapping, and sometimes hairy and glabrescent individuals occur in the same locality [42] (p. 498; NAP!); so, some populations throughout the Apennines, albeit attributable to one of the two taxa, show intermediate features [33]. On account of these considerations, of the overlapping geographical ranges, and, above all, of the molecular results, we chose to treat the two taxa as conspecific, but distinct at subspecific rank. Regarding the genetic distinctiveness of the ‘Italian clandestina’ from the ‘Balkan clandestina’, we refrain from proposing a split treatment of these taxa, as the only diagnostic feature hitherto found (i.e., the ratio between the cauline leaves and the internodes) is rather weak and not statistically investigated. We think that further morphometric, karyological, and molecular studies, with a more extensive sampling, might shed light on this critical issue.

Surprisingly, the same holds for *Mc. moraldoi*, which is nested in subsp. *rosanoi* in both nuclear and chloroplast analyses. Morphologically, *Mc. moraldoi* can be distinguished from *Mc. graminifolia* subsp. *rosanoi* especially on account of its elliptic cauline leaves and also by its more laxely casepitose habit and less rigid leaves. The latter might be possible adaptations to the different habitat (shadowy flysch rocks).

*Mcneillia graminifolia* subsp. *hungarica* has been traditionally associated to the Italian plants with densely hairy and not rigid leaves, i.e., subsp. *rosanoi* [27], or even regarded as not distinct from it (see Table 1). According to the nuclear tree, as said above, there is some affinity between subsp. *hungarica* and the Italian clade, likely due to the persistence of “ancestral” characters (see above). However, on one hand, the Romanian populations constantly differ from subsp. *rosanoi* by their shorter glandular hairs and petals roughly equalling the sepals [33]; on the other hand, they are reproductively isolated, because not only are their ranges sharply disjunct but are separate by the ‘Balkan clandestina’. In addition, the plastid tree clearly does not recover any particular affinity with the Italian taxa, but rather with the geographically closer *Mc. saxifraga* subsp. *saxifraga*. Therefore, we consider the specific level the most appropriate.

*Mcneillia pseudosaxifraga*, albeit described by Mattfeld [27] as a subspecies of *Mc. stellata* (but with some features resembling *Mc. saxifraga*) is regarded as a very distinct species by Halliday [29], “perhaps more closely related” to *Mc. saxifraga* as well. Interestingly, a relationship with both taxa is suggested by the plastid network (Figure 4), which indicates that the haplotype of *Mc. pseudosaxifraga* originated by a haplotype shared by *Mc. saxifraga* subsp. *tmolea* and a specimen of *Mc. stellata*. The monophyly of *Mc. pseudosaxifraga* is evident both in the nuclear and the chloroplast trees, and it is well supported by its morphological features [29,33], in some cases autapomorphic (i.e., the stems without dead leaves). As a consequence, we regard the specific rank as appropriate.

Regarding *Mc. stellata*, a species reported across Greece and Southern Albania, samples from the southern part of the range are different from the ones collected in the Pindhos massif. In particular, in the nuclear tree the northern individuals are included in a different, more internal clade as compared to the samples of *Mc. stellata* from the south. As in the case of ‘Italian clandestina’, the northern populations of *Mc. stellata* could be regarded as the result of a contact, presumably with the ‘clandestina’ lineage. This is suggested by the fact that a single specimen from Pindhos (APP no. 61429) is found close to ‘Balkan clandestina’ in plastid data. Indeed, the southern-most sampled individuals belong to the most typical form of the species (concurring with the lectotype). Whereas *Mc. stellata* is rather uniform throughout the central and southern sectors of its range, in the north it shows some morphological differences (not always correlated), such as the glabrous pedicels [28] and the longer leaves [30]. Incidentally, we note that these features somehow resemble those of *Mc. graminifolia* subsp. *clandestina*. For these reasons, these populations could be suspected to be of hybrid origin. They were described as var. *epirota* Halácsy [43] (p. 238), a taxon consistently disregarded in time [28,33], but possibly deserving subspecific rank, also in consideration of our molecular results.

## 4. Taxonomic Treatment

The articles cited throughout the paragraph follow the *Shenzen Code* [44] (hereafter ICN). For the names lectotypified here, we provide further details and links to images.

***Mcneillia*** Dillenb. & Kadereit{xe “Mcneillia Dillenb. & Kadereit”} in Taxon 63: 78. 2014—Type: *Mcneillia graminifolia* (Ard.) Dillenb. & Kadereit (≡ *Arenaria graminifolia* Ard.), selected by Dillenberger & Kadereit in Taxon 63: 83. 2014 *≡ Pettera* Rchb.{xe “Pettera Rchb.”}, Icon. Fl. Germ. Helv. 5: 33. 1841, nom. reji. (earlier homonym of *Petteria* C.Presl in Abh. Königl. Böhm. Ges. Wiss., ser. 5, 3: 569) (Art. 53.1 of ICN) (for the rejection of the name and its type, see ICN: App. III) ≡ *Minuartia* sect. *Lanceolatae* ser. *Graminifoliae* Mattf. in Repert. Spec. Nov. Regni Veg. Beih. 15: 130. 1922, type designated by McNeill in Notes Roy. Bot. Gard. Edinburgh 24: 143. 1962.

***Mcneillia graminifolia*** (Ard.) Dillenb. & Kadereit in Taxon 63: 83. 2014 ≡ *Arenaria graminifolia* Ard., Animadv. Bot. Spec. Alt. 25. 1764 (basion.) ≡ *Alsine graminifolia* (Ard.) J.F.Gmel., Syst. Nat. 2, ed. 13[bis]: 507. 1791 ≡ *Sabulina graminifolia* (Ard.) Rchb., Fl. Germ. Excurs. 2: 789. 1832 ≡ *Pettera graminifolia* (Ard.) Rchb., Icon. Fl. Germ. Helv. 5: 33. 1841 ≡ *Minuartia graminifolia* (Ard.) Jáv. in Sched. Fl. Hung. Exsicc. 2: 22. 1914—Type (lectotype): [Italy,] s. d., s. c., Herb Linn. no. 585.51 (LINN!, “no. 25. *Arenaria graminifolia* Arduino, Spec. 2 t. 8.”) (sent by Arduino himself in June 1763 to Linnaeus: http://urn.kb.se/resolve?urn=urn:nbn:se:alvin:portal:record-231713 (accessed on 26 July 2022); http://urn.kb.se/resolve?urn=urn:nbn:se:alvin:portal:record-231952) (accessed on 26 July 2022), designated by Conti & Santangelo in Taxon 50: 193. 2001.

= Arenaria arduinoi var. italica Vis., Stirp. Dalmat. Spec.: 8. 1826 ≡ Minuartia graminifolia [“Rasse”] italica (Vis.) Graebn. in Ascherson & Graebner, Syn. Mitteleur. Fl. 5(1): 762. 1918 ≡ Minuartia graminifolia subsp. rosanoi var. italica (Vis.) Mattf. in Beibl. Bot. Jahrb. Syst. 126: 31. 1921. Type: Not designated.

*Note:* Bluff et al. [45] published the combination *Alsine graminifolia* Bluff, Nees & Schauer, Comp. Fl. German. 1(2): 96. 1837, nom. illeg., non *Alsine graminifolia* J.F.Gmel., Syst. Nat. ed. 13[bis]: 507. 1791 (Art. 53.1 of ICN), citing *Eremogone graminifolia* Fenzl, Vers. Darstell. Alsin.: 37. 1833. This latter name was intended as based on the illegitimate *Arenaria graminifolia* Schrad., Hort. Gott. 1: 5. 1809, a later homonym of *Arenaria graminifolia* Ard. (Arts. 53.1 and 58.1 of ICN). *Eremogone graminifolia* is a synonym of *Eremogone saxatilis* (L.) Ikonn., according to POWO [31]. This latter database, however, incorrectly reports the combination by Bluff et al. [45] as “*Alsine graminifolia* (Ard.) Bluff, Nees & Schauer”, and therefore it is wrongly reported as a further synonym of *Mc. graminifolia* (i.e., *Alsine arduinoi* Fenzl according to Bluff et al. [45]).

− “*Alsine graminifolia* var. *hirsuta*” Vis., Fl. Dalmat. 3: 178. 1852, nom. inval. (Art. 26.2 of ICN).

*Note:* As Visiani [46] explicitly includes in this variety also the nomenclaturally typical elements of the species, i.e., *Arenaria graminifolia*, his combination is invalid.

− “*Alsine graminifolia* var. *typica*” Beck in Ann. Naturhist. Hofmus. 6: 324. 1891, nom. inval. (Art. 24.3 of ICN).

*Note:* Beck von Mannagetta [47] included in this latter name, both *Mc. graminifolia* and *Mc. rosanoi* subsp. *rosanoi*.

***Mcneillia hungarica*** (Jáv.) F.Conti & Del Guacchio, **comb. nov.** ≡ *Minuartia graminifolia* subsp. *hungarica* Jáv., Sched. Fl. Hung. Exsicc. 2: 22. 1914 (basion.) ≡ *Minuartia graminifolia* [“Rasse”] *hungarica* (Jáv.) Graebn. in Ascherson & Graebner, Syn. Mitteleur. Fl. 5(1): 764. 1918 ≡ *Minuartia graminifolia* subsp. *rosanoi* var. *hungarica* (Jáv.) Mattf. in Repert. Spec. Nov. Regni Veg. Beih. 15: 134. 1922. ≡ *Mcneillia graminifolia* subsp. *hungarica* (Jáv.) F.Conti & Bartolucci in Willdenowia 44: 289. 2014—Type (lectotype): [Romania,] “Comit. Krassó-Szörény. In fissuris rupium perpendicularium montis Arzsána supra pag. Ekés (olim Plugova) et Mehádia. Solo calc. Alt. ca. 1450 m”, 13.VII.1912, *S. Jávorka* (BP!), designated by Kováts in Ann. Hist. Nat. Mus. Nat. Hung. 92: 26. 2000; isolectotypes at BM (barcode BM000613243, n. v.), E (barcode E00318146, digital image!, available at https://data.rbge.org.uk/search/herbarium/?cfg=fulldetails.cfg&specimen_num=325647, accessed on 26 July 2022), G!, CL!, L (barcode L1711453, digital image!, available at https://data.biodiversitydata.nl/naturalis/specimen/L.1711453, accessed on 26 July 2022), US (barcode US 1346829, digital image!, available at http://n2t.net/ark:/65665/35f1be471-3541-4fb4-9895-f5b1d69d811f, accessed on 26 July 2022).

*Note:* Even if lacking the words “designated here” or an equivalent, the lectotype designation by Kováts [48] was effective, because proposed before 1 January 2001 (Art. 7.11 of *ICN*), and therefore predates that by Conti [33].

***Mcneillia pseudosaxifraga*** (Mattf.) Dillenb. & Kadereit in Taxon 63: 84. 2014 ≡ *Minuartia stellata* subsp. *pseudosaxifraga* Mattf. in Repert. Spec. Nov. Regni Veg. Beih. 15: 136. 1922 (basion.) **≡** *Minuartia pseudosaxifraga* (Mattf.) Greuter & Burdet in Willdenowia 12: 188. 1982—Type (lectotype): [Greece,] “m. Papingon et Gamila, Cepelovon Vradeton distr. Zagorion”, 13.VII.1896, *A. Baldaccii, Iter Albanicum (Epiroticum) IV n. 161* (B, n. v.), designated by McNeill in Notes Roy. Bot. Gard. Edinburgh 24: 341. 1963 (as holo-, cf. Art. 9.10 of ICN, destroyed, see below); (substitute lectotype, **designated here** by F. Conti & E. Del Guacchio, Art. 9.11 of ICN): [Greece,] “In rupestr. alp. [= on the rocky alpine places of] m. Papingon et Gamila, distr. Zagorion (Vradeton)”, 13.VII.1896, *A. Baldacci*, *Iter Albanicum (Epiroticum) IV n. 161* (G, barcode G00226886, sub *Alsine stellata*!, image available at https://www.ville-ge.ch/musinfo/bd/cjb/chg/adetail.php?id=200477&base=img&lang=fr, accessed on 7 June 2022); isolectotypes at BR (barcode 000000695411, digital image!, available at https://www.botanicalcollections.be/specimen/BR0000006954116, accessed on 7 June 2022), G (barcode G00226876!, image available at https://www.ville-ge.ch/musinfo/bd/cjb/chg/adetail.php?id=200476&base=img&lang=fr, accessed on 7 June 2022), K (barcode K000568026, digital image!, available at http://www.kew.org/herbcatimg/293420.jpg, accessed on 7 June 2022), and WU (nos. WU0074783!, image available at https://iiif.jacq.org/viewer/?manifest=https://services.jacq.org/jacq-services/rest/iiif/manifest/458429, accessed on 7 June 2022, and WU0074784, Herbarium Halácsy!, image available at https://iiif.jacq.org/viewer/?manifest=https://services.jacq.org/jacq-services/rest/iiif/manifest/458431, accessed on 7 June 2022).

*Note:* Mattfeld [27] validly published the name *Minuartia stellata* subsp. *pseudosaxifraga* providing a synonymy (the misapplied name “*Alsine graminifolia* Halacsy, Consp. Fl. Graec. I, 1901, 237, non Gmel.”), syntypes (“Exs.: Baldacci, Iter Alban. Epir. IV, no. 161”), a Latin description, and two localities (“Mt. Papingon und Gamila”, directly derived from the syntypes’ labels). McNeill [28] first proposed a lectotype, indicating a specimen at B as the “holotype” and another one in the Halácsy’s Herbarium at “W” (probably an error for WU) as isotype. However, he did not see the Berlin specimen and indeed he suggested that it might have been destroyed. Later, Strid [30] reported a mere citation of the syntypes’ labels and a list of herbaria in which they were preserved (“G!, K!, WU!, WU-Hal!”) (cf. also [49] (p. 62); Kamari [36] (p. 191)). Unfortunately, as the specimen at B was destroyed (“†”) [36], probably during World War II (see e.g., [50]), a new lectotype (among the extant syntypes) may be chosen (Art. 9.11 of ICN). Even if we regard the specimens at K and WU as isolectotypes as well as those at BR, G, they slightly differ for the dates reported on the labels. Namely, they report “13.25 Julio”, and might indeed not belong to the same gathering. Therefore, despite the specimens in WU being personally examined by Mattfeld, we choose as lectotype a sheet from the Herbarium Delessert at G, i.e., G00226886, because the label data concur with those provided by Mattfeld [27] (especially for the name reported by Baldacci and the locality data), the specimen is complete of three several well-preserved flowering individuals, and they were collected at the same place on the same date and so represent a single gathering (cf. Arts. 8.2 and 9.17 of ICN). The diagnostic features of the taxon are easily observable and therefore the specimen fully supports the current use of the name: height more than 2 cm, caespitose and lax habit, leaves narrowly lanceolate, greyish-green, densely glandular, and not rigid, stems rather woody, elongated and stout but without dead leaves, inflorescences with up to five flowers, petals up to ½ longer than sepals (see the leftmost individual), bracts narrow, lanceolate, and not scarious on margins.

***Mcneillia rosanoi*** (Ten.) F.Conti & Del Guacchio, **comb. nov.** ≡ *Arenaria rosanoi* Ten., Fl. Napol. 1 (Prodr.): XXVI. 1811 (basion., sub *Arenaria Rosani*, see Art. 60.8 of ICN).

***Mcneillia rosanoi*subsp. *clandestina*** (Port.) Del Guacchio & F.Conti, **comb. nov.** ≡ *Arenaria clandestina* Port., Enum. Pl. Dalmatia: 13. 1824 (basion.) ≡ *Alsine clandestina* (Port.) A.Kern., Sched. Fl. Exs. Austro-Hung. 2: 86 (n. 567). 1883 [“1882”] ≡ *Alsine graminifolia* subsp. *clandestina* (Port.) Wettst. in Biblioth. Bot. 5(26): 36. 1892 ≡ *Alsine graminifolia* var. *clandestina* (Port.) Beck in Glasn. Zemaljsk. Muz. Bosni Hercegovini 18: 492. 1906 ≡ *Minuartia graminifolia* subsp. *clandestina* (Port.) Mattf. in Bot. Jahrb. Syst. 57(2, Beibl. 126): 31 ≡ *Minuartia clandestina* (Port.) Trinajstić, Suppl. Fl. Anal. Jugosl. 5: 6. 1978 ≡ *Mcneillia graminifolia* subsp. *clandestina* (Port.) Dillenb. & Kadereit, in Taxon 63: 83. 2014—Type (neotype): [Republic of Croatia,] “Dalmatia”, I.1713(?), *Vis. [= R. Visiani] s. n.* (PAD, sub *Arenaria Arduini*, the rightmost individual!), designated by Conti in Bot. J. Linn. Soc. 143: 426. 2003.

= *Arenaria arduinoi* Vis., Stirp. Dalmat. Spec.: 8. 1826 (sub *A. arduini*, Art. 60.8 of ICN), nom. illeg. (Art. 52.2 of ICN) ≡ *Alsine arduinoi* (Vis.) Fenzl, Vers. Darstell. Alsin.: 57 (in tab.). 1833—Type (lectotype): [Republic of Croatia,] “E monti Biokovo [= from Mt. Biokovo] in Dalmatia”, IX s. a., *[R.] Visiani* s. n. (G!, individual in the middle), designated by Conti in Bot. J. Linn. Soc. 143: 426. 2003.

*Note:* As Visiani [51] includes in *Arenaria arduinoi* also the previous and legitimate *Arenaria graminifolia* by Arduino, his name is superfluous and illegitimate.

= *Arenaria arduinoi* var. *dalmatica* Vis., Stirp. Dalmat. Spec.: 8. 1826 (sub *A. Arduini* var. *dalmatica*) ≡ *Alsine rosanoi* var. *dalmatica* (Vis.) Guss., Fl. Sicul. Syn. 1: 498. 1843 (“1842”) ≡ *Alsine graminifolia* var. *dalmatica* Beck in Ann. Naturhist. Hofmus. 6: 323. 1891—Type: Not designated.
= *Arenaria alpicola* Ten., Fl. Napol. 4: 224. 1830—Type (lectotype): [Italy,] “Monte dei Fiori, Pizzo di Sivo, Majella | Costone nella discesa verso il Campiglione”, s. d., s. c., s. n. (NAP, barcode NAP000259-B!, sub *Arenaria Rosani*, *A. alpicola*), designated by Conti & Santangelo in Taxon 50: 193. 2001.
= *Alsine graminifolia* var. *glaberrima* Vis., Fl. Dalmat. 3: 178. 1852 ≡ *Minuartia graminifolia* var. *glaberrima* (Vis.) Hayek in Denkschr. Akad. Wiss. Wien, Math.-Naturwiss. Kl. 94: 135. 1917—Type: Not designated.
= *Alsine graminifolia* var. *semiglabra* Vis., Fl. Dalmat. 3: 178. 1852—Type: Not designated.
= *Alsine graminifolia* var. *dinarica* Beck in Ann. Naturhist. Hofmus. 6: 324. 1891 ≡ *Minuartia clandestina* f. *dinarica* (Beck) Trinajstić, Suppl. Fl. Anal. Jugosl. 5: 6. 1978—Type: Not designated.
= *Alsine graminifolia* var. *dinarica* f. *subglabra* Beck in Ann. Naturhist. Hofmus. 6: 324. 1891 ≡ *Minuartia graminifolia* [“Rasse”] *clandestina* var. *dinarica* f. *subglabra* (Beck) Graebn. in Ascherson & Graebner, Syn. Mitteleur. Fl. 5(1): 763. 1918 ≡ *Minuartia clandestina* f. *subglabra* (Beck) Trinajstić, Suppl. Fl. Anal. Jugosl. 5: 6. 1978—Type: Not designated.
− “*Arenaria rosanoi* var. *subglabra*” Ten., Syll. Pl. Fl. Neapol.: 218. 1831 is a nom. nud. (Art. 38.1 of ICN) (cf. [33]). It was otherwise published by Tenore [52] (p. 224) as “*A. Rosani* var. B. *glabriuscula*”, but in synonymy (Art. 36.1(b) of ICN).

***Mcneillia rosanoi*subsp. *moraldoi*** (F.Conti) Del Guacchio & F.Conti, **comb. nov. et stat. nov.** ≡ *Minuartia moraldoi* F.Conti, in Plant Biosyst. 135: 193. 2001 (basion.) ≡ *Mcneillia moraldoi* (F.Conti) Dillenb. & Kadereit in Taxon 63: 84. 2014—Type (holotype): [Italy,] “Campania, Cilento (SA), versante occidentale del M. Sacro o Gelbison, rupi di flysch del Cilento nella faggeta, 1650 m”, 21.VI.1999, *F. Conti & A. Alessandrini s. n.* (FI, barcode FI001221!); isotypes at NAP (barcode NAP0000265!) and APP (ex Herb. Conti, no. 1628!).

***Mcneillia rosanoi*subsp. *rosanoi*** ≡ *Arenaria rosanoi* Ten., Fl. Napol. 1 (Prodr.): XXVI. 1811 ≡ *Alsine rosanoi* (Ten.) Guss., Fl. Sicul. Syn. 1: 498. 1843 ≡ *Alsine graminifolia* var. *rosanoi* (Ten.) Bég., Exsicc. (Fl. Ital.), ser. 2: no. 1450. 1911 ≡ *Minuartia graminifolia* subsp. *rosanoi* (Ten.) Mattf. in Beibl. Bot. Jahrb. Syst. 126: 31. 1921 ≡ *Mcneillia graminifolia* subsp. *rosanoi* (Ten.) F.Conti, Bartolucci, Iamonico & Del Guacchio, Phytotaxa 170: 139. 2014—Type (lectotype): [Italy,] “Basilicata”, s. d., *[F. Rosano] s. n.* (NAP, barcode NAP0000263!), designated by Conti & Santangelo in Taxon 50: 195. 2001.

***Mcneillia saxifraga*** (Friv.) Dillenb. & Kadereit, Taxon 63: 84. 2014 ≡ *Arenaria saxifraga* Friv. in Flora 19: 434. 1836 (basion.) ≡ *Alsine saxifraga* (Friv.) Boiss., Diagn. Pl. Orient. 1: 47 1843 ≡ *Minuartia saxifraga* (Friv.) Graebn. in Ascherson & Graebner, Syn. Mitteleur. Fl. 5(1): 756. 1918—Type (lectotype): [Bulgaria,] “In Rumelia”, 1835, *[I.] Frivaldszky s. n.* (BP, n. v.), designated by McNeill in Notes Roy. Bot. Gard. Edinburgh 24: 340. 1963 (as holo-, cf. Art. 9.10 of ICN); isolectotype at G-BOIS (?, n. v., cf. Strid 1986).

*Notes:* Apparently, the lectotype proposed by McNeill (1963), and cited by Strid (1986) and Kamari (1997) was never seen by any of them, and not even by us. Besides, the presumed isolectotypes at CAS and K cited by these authors, as other material linked to Frivaldszky and located by us, were collected after the protologue publication or do not report any date at all: CAS (barcode CAS0027648, digital image!, available at https://plants.jstor.org/stable/viewer/10.5555/al.ap.specimen.cas0027648, accessed on 18 June 2022), HAL (barcode HAL0118108, digital image!, available at https://plants.jstor.org/stable/viewer/10.5555/al.ap.specimen.hal0118108, accessed on 18 June 2022), K (barcodes K000568070, digital image!, available at http://specimens.kew.org/herbarium/K000568070, accessed on 18 June 2022, and K000568071, digital image!, available at http://specimens.kew.org/herbarium/K000568071 accessed on 18 June 2022).

− “*Minuartia saxifraga* subsp. *rumelica*” Mattf. in Bot. Jahrb. Syst. 57(2, Beibl. 126): 31, nom. inval. (Art. 26.2 of ICN).

*Note:* As Mattfeld [27,53] explicitly includes in this infraspecific taxon the nomenclaturally typical element of the species, i.e., *Arenaria saxifraga*, his combination is invalid.

***Mcneillia stellata*** (E.D.Clarke) Dillenb. & Kadereit, Taxon 63: 84. 2014 ≡ *Cherleria stellata* E.D.Clarke, Travels Eur. Asia & Africa 2(3): 211. 1816 (basion.) ≡ *Alsine stellata* (E.D.Clarke) Halácsy, Denkschr. Kaiserl. Akad. Wiss., Wien. Math.-Naturwiss. Kl. 61: 232. 1894 ≡ *Minuartia stellata* (E.D.Clarke) Maire & Petitm. in Matér. Étude Fl. Géogr. Bot. Orient 4: 48. 1908 ≡ *Arenaria stellata* (E.D. Clarke) Fernald, Rhodora 21: 6. 1919—Type (lectotype): [Greece,] “Mt. Parnassus”, 16.XII.1801, [E. D.] *Clarke s. n.* (BM, barcode BM00061324, digital image! available at https://data.nhm.ac.uk/object/3644b737-8c68-4f01-82f7-4c3e573cf77b/1659916800000, accessed on 18 June 2022), designated by Strid, Mountain Fl. Greece 1: 100. 1986 (as “type”).

*Note:* The type indication by McNeill [28] (p. 342, “holo. BM?”) is hardly considerable as an effective lectotypification (J. McNeill, pers. comm.). In fact, Art 7.11 requires that “for purposes of priority (Arts. 9.19, 9.20, and 10.5 of ICN), designation of a type is achieved only if the type is definitely accepted as such by the typifying author”. As McNeill [28] was uncertain that the specimen was in BM, it is not clear that he could be held to have definitely accepted it. Therefore, we prefer to rely on the statement by Strid [30].

***Mcneillia tmolea*** (Mattf.) F.Conti & Del Guacchio, **comb. nov. et st. nov.** ≡ *Minuartia saxifraga* subsp. *tmolea* Mattf. in Repert. Spec. Nov. Regni Veg. Beih. 15: 132. 1922 (basion.) ≡ *Mcneillia saxifraga* subsp. *tmolea* (Mattf.) Dillenb. & Kadereit, in Taxon 63: 84. 2014—Type (lectotype): [Turkey,] “Sommet du Tmolus, au-dessus de l’Yaila de Bozdagh”, 19.VII.1854, *B. Balansa pl. D’Orient n. 112* (B, sub *Alsine saxifraga*, n.v.), designated by McNeill in Notes Roy. Bot. Gard. Edinburgh 24: 341. 1963 (as holo-, cf. Art. 9.10 of ICN); (substitute lectotype, **designated here** by F. Conti & E. Del Guacchio, Art. 9.11 of ICN): [Turkey,] “Sommet du Tmolus, au-dessus de l’Yaila de Bozdagh”, 19.VII.1854, *B. Balansa pl. D’Orient n. 112* (JE, barcode JE00009377!, sub *Alsine saxifraga* Boiss., image available at https://je.jacq.org/JE00009377, accessed on 26 July 2022); isolectotypes at BM (barcode BM000946399!, image available at https://data.nhm.ac.uk/dataset/collection-specimens/resource/05ff2255-c38a-40c9-b657-4ccb55ab2feb/record/471663, accessed on 26 July 2022), GOET (barcode 000617!, image available at https://plants.jstor.org/stable/viewer/10.5555/al.ap.specimen.goet000617, accessed on 26 July 2022), JE (barcodes JE00009377, digital image!, available at https://www.jacq.org/detail.php?ID=150570, accessed on 26 July 2022, JE00009378, digital image!, available at https://je.jacq.org/JE00009378, accessed on 26 July 2022, and JE00009379, digital image!, available at https://je.jacq.org/JE00009379, accessed on 26 July 2022), K (barcodes K000395883!, image available at http://specimens.kew.org/herbarium/K000395883, accessed on 26 July 2022 [the rightmost individual is very aberrant and possibly *Mc. saxifraga*], and K000395884!, image available at http://specimens.kew.org/herbarium/K000395884, accessed on 26 July 2022), L (barcodes L984920 (n. v.), and L221040 (n. v.), fide [26]), MEL (barcode MEL2504100, digital image!, available at https://plants.jstor.org/stable/viewer/10.5555/al.ap.specimen.mel2504100, accessed on 26 July 2022), P (barcodes P04990991, digital image!, available at https://www.gbif.org/occurrence/667424286, accessed on 26 July 2022, P04990992, digital image!, available at https://www.gbif.org/occurrence/667424285, accessed on 26 July 2022, and P04990993, digital image!, available at https://www.gbif.org/occurrence/667424284, accessed on 26 July 2022), and WAG (barcode 0004040, digital image!, available at https://plants.jstor.org/stable/viewer/10.5555/al.ap.specimen.wag0004040, accessed on 26 July 2022). Other syntypes: [Turkey,] “Boz Dagh, rupes Tmoli supra Bozdagh”, VI.1852, *[E.] Boissier s. n.* (BM, barcode BM000946400!, image available at https://data.nhm.ac.uk/dataset/collection-specimens/resource/05ff2255-c38a-40c9-b657-4ccb55ab2feb/record/471664, accessed on 26 July 2022; G?, cf. [28]; JE, barcode JE00009380!, image available at https://je.jacq.org/JE00009380, accessed on 26 July 2022); GOET, barcodes 000554, n. v., 006008, n. v., fide [26], and 000616, digital image!, available at https://plants.jstor.org/stable/viewer/10.5555/al.ap.specimen.goet000616, accessed on 26 July 2022); JE, barcode JE00009380, digital image!, available at https://plants.jstor.org/stable/viewer/10.5555/al.ap.specimen.je00009380, accessed on 26 July 2022; K, barcode K000395885!, image available at http://specimens.kew.org/herbarium/K000395885, accessed on 26 July 2022); LIVU, n. v. (fide [37]); P, barcodes P04990995, digital image!, available at https://www.gbif.org/occurrence/667424282, accessed on 26 July 2022, and P04990997 [only the individuals on the left!] digital image!, available at https://www.gbif.org/occurrence/667424280, accessed on 26 July 2022); US, barcodes 03617105, digital image!, available at https://www.gbif.org/occurrence/2452348481, accessed on 26 July 2022, and 03617106, digital image!, available at https://www.gbif.org/occurrence/2452293988, accessed on 26 July 2022). The following sheets at P include plants belonging to the gatherings by both Balansa and Boissier: P04990994 (digital image!, available at https://www.gbif.org/occurrence/667424283, accessed on 26 July 2022), P04990996 (digital image!, available at https://www.gbif.org/occurrence/667424281, accessed on 26 July 2022).

− *“Minuartia saxifraga* subsp. *tmolea”* Mattf. in Bot. Jahrb. Syst. 57(2, Beibl. 126): 31, nom. inval. (Art. 38.1 of ICN).

*Notes:* The name *Minuartia stellata* subsp. *tmolea* appeared for the first time in [53], but without any diagnosis or description (invalid name, cf. Art. 38.1 of ICN). In December, Mattfeld [27] validly published the new subspecies by a diagnosis in the key at p. 132, also providing taxonomic notes at p. 133 with syntypes from Mt. Tmolus (*locus classicus atque unicus*): (1) a specimen or specimens by Boissier, without any indication of herbarium, and the no. 122 of Balansa’s gathering in the herbarium of Haussknecht. McNeill [28] reported several syntypes of two gatherings, listing, under that by Balansa: “holo. B (destroyed?); iso. BM!, G, JE!, K!”. In a successive treatment [37], it becomes clear that at BM, JE, and K specimens of both the gatherings are present, but the “holotype” in B is cited no more. In addition, McNeill located a further specimen of Boissier’s gathering at LIVU. McNeill [28] chose a specimen by Balansa from B, although Mattfeld [27] explicitly cited the Haussknecht’s herbarium, which has been kept at JE since the times of his owner [54]. In fact, as reported above, two duplicates by Balansa are preserved in JE, and these are the only ones traced by us as belonging to Haussknecht’s herbarium. However, as the Art. 9.12 of ICN does not provide any preference between syntypes and isosyntypes in lectotypification designation, the choice by McNeill [28] is correct. Nevertheless, as the previous lectotype at B is unavailable, a new lectotype may be designated (Art. 9.11 of ICN). In this case, it seems appropriate to propose another syntype of the series already chosen by McNeill [28], i.e., *Balansa n. 112*, and namely in the Haussknecht herbarium at JE. There we traced two duplicates: barcodes JE00009377 (also included in the Herbarium Gaillardot), and JE 00009379 (with a print label). Both specimens were revised by Mattfeld, and the former also seen by McNeill, as indicated by modern labels. They include several fruiting individuals (fragments?). JE00009377 is more complete and shows the diagnostic features of the taxon: few-flowered inflorescences not more than 2 cm long (but cf. also P04990995!), cauline leaves strictly lanceolate with parallel veins. Other specimens belonging to the same gathering, but not marked as “*Balansa n. 112*” (e.g., JE00009378) are nevertheless regarded here as syntypes (Art. 8.2 of ICN). In addition to the syntypes reported by previous authors, we located abundant material elsewhere.

***Minuartiella brachypetala*** (Kamari) P.Caputo, D.De Luca, Iamonico, F.Conti & Del Guacchio **comb. nov. et st. nov.** ≡ *Minuartia graminifolia* subsp. *brachypetala* Kamari in A. Strid & Kit Tan (eds.), Fl. Hellenica 1: 190. 1997 (basion.) ≡ *Mcneillia graminifolia* subsp. *brachypetala* (Kamari) Dillenb. & Kadereit in Taxon 63: 83. 2014—Type (holotype): “Greece, W Macedonia. Nom. Florinis: Mt. Boutsi, summit area, south-west of the village of Vatochorion (9 km along the forest road), 1650–1750 m”, 8.VII.1981, *Strid* et al. *n. 18743* (C!; iso-B!, EGE, UPA).

## 5. Materials and Methods

### 5.1. Sample Collection and DNA Extraction

We collected leaf material from 29 herbarium specimens of *Mcneillia* (Herbarium codes according to Thiers [55]), representing all known taxa across their whole range (Table 2, Figure 1).

**Figure 5:**
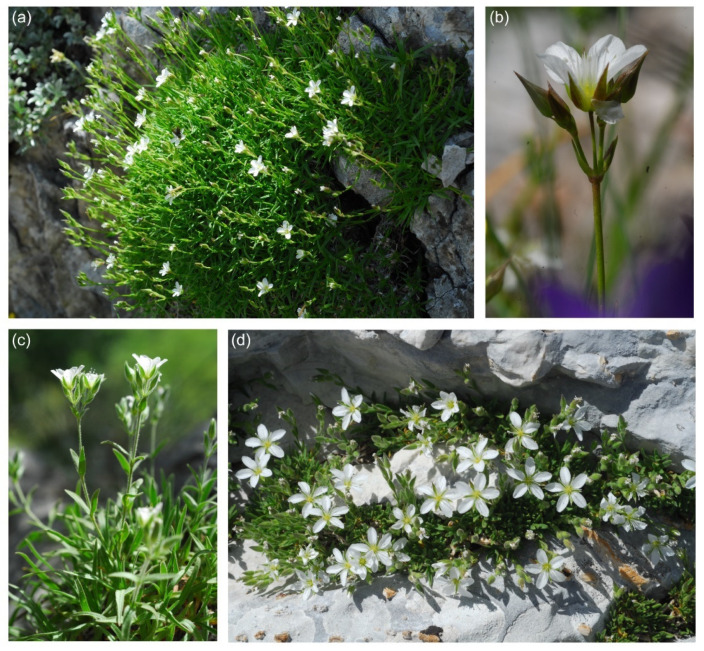
Photographs of some *Mcneillia* taxa observed in nature. (**a**) *Mc. graminifolia* subsp. *clandestina* (Pizzo Cefalone, Italy); (**b**) *Mc. graminifolia* subsp. *clandestina*, detail of the flowering stem (Montagna dei Fiori, Italy); (**c**) *Mc. graminifolia* subsp. *rosanoi* (Mt. Briccialone, Italy); (**d**) *Mc. pseudosaxifraga* (Mt. Nemercka, Albania). Photo credit: F. Conti.

In this regard, we relied on the above-mentioned taxonomic treatment in POWO [31], which is the most comprehensive, to verify the taxonomic value of critical taxa, such as *Mc. graminifolia* subsp. *hungarica* and subsp. *rosanoi*. As the outgroup, we chose one specimen of *Mn. recurva* (All.) Schinz & Thell. subsp. *condensata* (C.Presl) Greuter & Burdet, selected according to both its position in the molecular phylogeny by Dillenberger and Kadereit [26] and availability (Table 2). An illustration of some *Mcneillia* taxa is provided in Figure 5.

Total DNA was extracted using the GeneAll^®^ Exgene™ Plant SV mini kit (GeneAll Biotechnology, Seoul, Korea) following the manufacturer’s protocol for dried material. Plant material was grinded to powder using Mixer Mill 300 (Retsch^®^, Verder Scientific, Haan, Germany). The quality and quantity of extracted DNA was evaluated by 0.8% gel electrophoresis using the high-molecular weight marker HyperLadder™ 1 Kb (Bioline, Meridian Bioscience, Cincinnati, OH, USA).

### 5.2. Marker Selection, Amplification, and Sequencing

We selected eight molecular markers: two from the nuclear (ETS and ITS regions) and six from the chloroplast genome (*rpo*C1, *rps*16 intron, *rps*16-*trn*Q, *rpl*32-*trn*L, *trn*L-*trn*F, and *trn*H-*psb*A). These genomic regions were amplified by polymerase chain reaction (PCR) into a final volume of 25 µL containing: 7–10 ng DNA, 2X Kodaq PCR MasterMix (Applied Biological Materials Inc. ^®^, Richmond, BC, Canada), 400 nM forward and reverse primers, and water to volume. PCR conditions and primers are listed in Table S1. The amplification products were separated by 1.5% agarose gel electrophoresis in TBE 0.5×X buffer and visualised under UV light after staining with SafeView^™^ Classic (ABM^®^, Richmond, BC, Canada). The amplified products were purified using the NucleoSpin^®^ Gel and PCR Cleanup kit (Macherey-Nagel, Düren, Germany) and quantified on a 1.5% agarose gel. Sequencing reactions were carried out in a final volume of 5 µL using the BrightDye^®^ Terminator Cycle Sequencing Kit (MCLAB, Harbor Way, San Francisco, CA, USA), and purified using the the BigDye^®^ Xterminator^™^ Purification Kit (Applied Biosystems, Thermo Fisher Scientific, Foster City, CA, USA). The ITS, *rps*16 intron and *rps*16-*trn*Q markers were sequenced in both directions, while the others in one direction if the signal was unambiguous; for the intergenic spacer *rpl32*-*trn*L (over 1200 bp long), we sequenced only the variable region towards the 3′ end because of the lack of variation in other regions and the occurrence of long polynucleotide stretches that hampered the correct reading and assembling of reads. Capillary electrophoresis was carried out in the Applied Biosystems^®^ 3130 Genetic Analyzer (Applied Biosystems, Thermo Fisher Scientific, Foster City, CA, USA).

### 5.3. Sequence Alignment and Exploratory Data Analysis

Electropherograms were visually inspected for ambiguities, and then forward and reverse sequences were assembled in contigs before individually aligning them with the ClustalW algorithm [56] as implemented in the BioEdit v7.2.6 software [57]. Standard IUPAC ambiguity codes were used when base peaks overlapped, or the lower peak was at least one-third in height as the highest one. All sequences were deposited in GenBank/DDBJ (see Data availability section for details). Nuclear and chloroplast loci were then separately concatenated in two matrices using Mesquite v3.51 [58]. The best fitting evolution models were computed for each dataset in jModelTest v2.1.3 [59] using the corrected Akaike information criterion [60]. Exploratory Bayesian phylogenetic analyses were then individually carried out in MrBayes v3.2.6 [61] on both nuclear and chloroplast *Mcneillia* datasets, in two replica runs of four chains (one of which heated) for 1,000,000 generations, sampling chains every 1000 generations, under the default relaxed clock. Convergence and effective sample sizes (ESS) for all parameters were investigated in Tracer v.1.7 [62], the latter considered acceptable when >200. The first 10% of the samples was discarded as burn-in. The majority-rule consensus trees were visualized using FigTree v1.4.3 (http://tree.bio.ed.ac.uk/software/figtree/, accessed on 19 September 2021).

By observing branch lengths from the investigations above, the samples of *Mc. graminifolia* subsp. *brachypetala* resulted as surprisingly different from the rest of the ingroup. We therefore decided to further verify the phylogenetic position of the said taxon. To this aim, we integrated our ITS sequences with the 255 sequences employed by Dillenberger and Kadereit [26]. The sequences were de novo aligned and the resulting alignment was trimmed at the same length of our sequences. After a Bayesian investigation, we discovered that *Mc. graminifolia* subsp. *brachypetala* was sister group to the representatives of genus *Minuartiella* (abbreviated as *Ml.* from now onwards) included in Dillenberger and Kadereit [26]. We therefore added the five *Minuartiella* ITS sequences published by Koç et al. [35] (MK089560.1 to MK089564.1, KF737436.1, and KF737437.1) to the previous dataset, for a total of 290 accessions.

A Bayesian analysis was then carried out in MrBayes on the said ITS dataset with two replica runs and four chains for 5,000,000 generations and sampling chains every 5000 under the JC + G model.

### 5.4. Analysis of the Final Datasets

The three accessions of *Mcneillia graminifolia* subsp. *brachypetala* were then removed from the *Mcneillia* datasets, which were re-aligned and subjected to a novel computation of the evolution model as indicated above, but in this case separately for each nuclear and chloroplast DNA region. Incongruence between the nuclear and chloroplast DNA was evaluated both by the observation of conflicting topologies with a posterior probability ≥ 0.99 [63] and by a formal incongruence length difference (ILD) test [64,65] carried out in PAUP* v4.0a build 169 [66] starting with 100 replications and increasing the search up to 10,000 trees. A test of the clockwise behaviour of each dataset was carried out with two stepping-stone analyses testing strict and relaxed clock models, run in MrBayes in two replicas, sampling 200 steps of 50,000 generations each.

Bayesian analyses were carried out again on both nuclear and chloroplast *Mcneillia* datasets with the above-mentioned software for 1,000,000 generations, sampling chains every 1000 generations, under a strict clock, evaluating the quality of run parameters and visualizing trees as indicated above. To evaluate the relationships among the chloroplast haplotypes, we inferred a TCS network [67] in the software PopART [68] using a parsimony threshold of 95% for the calculation of the statistical parsimony algorithm [69].

## Figures and Tables

**Table 1:** Synopsis of the most comprehensive treatments of *Mcneillia* [abbr. “*Mc.*”]/*Minuartia* sect. *Lanceolatae* ser. *Graminifoliae* [abbr. “*Mn.*”]. Authorships omitted for the sake of brevity. Legend: (-) not accepted, (X) not known/not treated, ^(1)^ included in the subsp. *graminifolia*.

POWO [31]	Dillenberger and Kadereit [26]	Marhold [34]	Conti [33]	Halliday [29]
*Mc. graminifolia* subsp. *brachypetala*	*Mc. graminifolia* subsp. *brachypetala*	*Mn. graminifolia* subsp. *brachypetala*	*Mn. graminifolia* subsp. *brachypetala*	X
*Mc. graminifolia* subsp. *clandestina*	*Mc. graminifolia* subsp. *clandestina*	*Mn. graminifolia* subsp. *clandestina*	*Mn. graminifolia* subsp. *clandestina*	*Mn. graminifolia* subsp. *clandestina*
*Mc. graminifolia* subsp. *graminifolia*	*Mc. graminifolia* subsp. *graminifolia*	*Mn. graminifolia* subsp. *graminifolia*	*Mn. graminifolia* subsp. *graminifolia*	*Mn. graminifolia* subsp. *graminifolia*
*Mc. graminifolia* subsp. *hungarica*	- ^(1)^	- ^(1)^	*Mn. graminifolia* subsp. *hungarica*	- ^(1)^
*Mc. graminifolia* subsp. *rosanoi*	- ^(1)^	- ^(1)^	*Mn. graminifolia* subsp. *rosanoi*	- ^(1)^
*Mc. moraldoi*	*Mc. moraldoi*	X	*Mn. moraldoi*	X
*Mc. pseudosaxifraga*	*Mc. pseudosaxifraga*	*Mn. pseudosaxifraga*	*Mn. pseudosaxifraga*	*Mn. pesudosaxifraga*
*Mc. saxifraga* subsp. *saxifraga*	*Mc. saxifraga* subsp. *saxifraga*	*Mn. saxifraga* subsp. *saxifraga*	*Mn. saxifraga* subsp. *saxifraga*	*Mn. saxifraga* subsp. *saxifraga*
*Mc. saxifraga* subsp. *tmolea*	*Mc. saxifraga* subsp. *tmolea*	*Mn. saxifraga* subsp. *tmolea*	*Mn. saxifraga* subsp. *tmolea*	*Mn. saxifraga* subsp. *tmolea*
*Mc. stellata*	*Mc. stellata*	*Mn. stellata*	*Mn. stellata*	*Mn. stellata*

**Table 2:** Taxa examined in the study. N = number of individuals sampled. * = classic locality.

Taxon	N	Voucher Specimen
*Mcneillia graminifolia* subsp. *brachypetala*	2	GREECE. Mt. Boutsi * (APP nos. 36275 and 36277).
	1	GREECE. Mt. Boutsi * (B, barcode B 10 0366304, isotype!).
*Mc. graminifolia* subsp. *clandestina*	1	CROATIA. Mt. Biokovo * (FI, barcode FI066602).
	1	CROATIA. Mt. Biokovo * (APP no. 58584).
	1	ITALY. Gran Sasso (APP no. 42385).
	1	ITALY. Montagna dei Fiori (APP no. 575).
	1	ITALY. Mt. Vettore (APP no. 62555).
	1	MONTENEGRO. Durmitor (B, barcode B 10 1092939).
*Mc. graminifolia* subsp. *graminifolia*	2	ITALY. Vette di Feltre * (APP nos. 42396 and 42397).
	1	ITALY. Vette di Feltre * (FI, barcode FI066605).
*Mc. graminifolia* subsp. *hungarica*	1	ROMANIA. Mt. Arjana *, Fl. Romaniae Exsicc. 1221 (RO).
	1	ROMANIA. Mt. Arjana *, Fl. Romaniae Exsicc. 1221b (RO).
*Mc. graminifolia* subsp*. rosanoi*	1	ITALY. Gran Sasso (APP no. 42436).
	1	ITALY. Mt. Secine (APP no. 42444).
	1	ITALY. Mt. Serrone (APP no. 42441).
	1	ITALY. Isnello (APP no. 42407).
*Mc. moraldoi*	1	ITALY. Mt. Gelbison * (NAP, barcode NAP0000265).
*Mc. pseudosaxifraga*	1	ALBANIA. Mt. Nemercka (APP no. 48873).
	1	GREECE. North Pindhos (APP nos. 36270).
	1	GREECE. Mt. Timphi (B, barcode B 10 1052716).
*Mc. saxifraga* subsp. *saxifraga*	1	BULGARIA. Balkan * (B, barcode B 10 1092946).
	1	GREECE. Mt. Kerkini, Greuter no. 16756 (C).
*Mc. saxifraga* subsp. *tmolea*	1	TURKEY. Mt. Bozdağ * (FI, barcode FI066608).
*Mc. stellata*	1	GREECE. Mt. Erimanthos, Strid no. 33496 (C).
	1	GREECE. Mt. Parnassos *, Baden & Franzen no. 940 (C).
	2	GREECE. Mt. Tsoumerka (APP nos. 43626 and 61429).
*Mn. recurva* subsp. *condensata*	1	ITALY. Mt. Volturino (APP no. 63095).

## Data Availability

All sequences are available at the following accession numbers: LC714779-LC714806 (ETS); LC714807-LC714836 (ITS); LC714914-LC714942 (*rps*16 intron); LC714943-LC714972 (*psb*A-*trn*H); LC714973-LC715002 (*trn*L-*trn*F); LC715003-LC715030 (*trn*L-*rpl*32); LC715031-LC715060 (*trn*Q-*rps*16); LC715061-LC715064 (rRNA pseudogenes); LC715065-LC715094 (*rpo*C1).

## Supplementary Materials

The following supporting information can be downloaded at: https://www.mdpi.com/article/10.3390/plants11162118/s1, Table S1: List of primers and amplification conditions for nuclear and chloroplast markers; Data S1: Nuclear alignment in nexus format; Data S2: Chloroplast alignment in nexus format. References [70,71,72,73,74,75,76,77,78] are cited in Supplementary Materials.
