# Supplementary material for: Relationships within Mcneillia Indicate a Complex Evolutionary History and Reveal a New Species of Minuartiella (Caryophyllaceae, Alsinoideae)
Source: Plants (Basel). 2022 Aug 15;11(16):2118. doi: 10.3390/plants11162118 (PMC9414604; doi:10.3390/plants11162118)
Supplement: Supplementary file 1 [file plants-11-02118-s001.zip › Table S1.pdf]

Table S1. List of primers and amplification conditions for nuclear and chloroplast markers.

Genome	Marker	Oligo name	Primer sequence (5'-3')	Reference	PCR program
nuclear	ETS	18S-2L	TGACTACTGGCAGGATCAACCAG	Linder et al. (2000)	B
		ETS-Lar	GGGAAACTTGTATATCGCGCCTAGTAGTCC	Moore & Kadereit (2013)	
	ITS	JK14	GGAGAAGTCGTAACAAGGTTTCCG	Aceto et al. (1999)	A
		JK12	CCAAACAACCCGACTCGTAGACAGC	Aceto et al. (1999)	
chloroplast	*rpo*C1	rpoC1_1	GTGGATACACTTCTTGATAATGG	Ford et al. (2009)	B
		rpoC1_3	TGAGAAAACATAAGTAAACGGGC	Ford et al. (2009)	
	*rps*16 intron	rpSF	GTGGTAGAAAGCAACGTGCGACTT	Popp & Oxelman (2001)	B
		rpSR2	TCGGGATCGAACATCAATTGCAAC	Popp & Oxelman (2001)	
	*rps*16-trnQ	rps16x1	GTTGCTTTYTACCACATCGTTT	Shaw et al. (2007)	B
		trnQ	GCGTGGCCAAGYGGTAAGGC	Shaw et al. (2007)	
	*rpl*32-*trn*L	trnL$^{(UAG)}$	CTGCTTCCTAAGAGCAGCGT	Shaw et al. (2007)	A
		rpl32-F	CAGTTCCAAAAAAACGTACTTC	Shaw et al. (2007)	
	*trn*L-*trn*F	trnF1(e)	GGTTCAAGTCCCTCTATCCC	Taberlet et al. (1991)	C
		trnF2(f)	ATTTGAACTGGTGACACGAG	Taberlet et al. (1991)	
	*trn*H-*psb*A	trnH2	CGCGCATGGTGGATTCACAATCC	Tate et al. (2002)	C
		psbAF	GTTATGCATGAACGTAATGCTC	Sang et al. (1997)	

Program	Initial denaturation	Denaturation	Annealing	Extension	Final extension	Notes
A	94°C, 3 min	94°C, 30 s	See note, 30 s	72°C, 1 min 30 s	7 min	T_a ITS = 62°C; T_a *rpl*32-*trn*L = 53°C;
		x 35 cycles				
B	94°C, 3 min	94°C, 30 s	See note, 30 s	72°C, 1 min	1 min	T_a ETS = 59°C; T_a *rpo*C1 and *rps*16 intron = 55°C; T_a *rps*16-*trn*Q = 53°C
		x 35 cycles				
C	94°C, 3 min	94°C, 30 s	55°C, 30 s	72°C, 35 s	1 min	
		x 35 cycles				
